# Promoting occupational health information in small and medium-sized enterprises in Japan

**DOI:** 10.1539/eohp.2024-0014

**Published:** 2025-03-18

**Authors:** Teppei Imai, Noriko Nishikido, Jiro Moriguchi, Sho Kondo, Hayato Terada, Akiko Saito, Eiji Shibata, Satoko Shimamoto, Yasuaki Jikumaru, Tamotsu Sugawara, Kuniaki Seiji, Masaji Tabata, Hiroto Nakadaira, Toshiyuki Hara, Mitsuo Hinoue, Izumi Matsumoto, Rie Narai, Hideyuki Takayama, Yoto Taguchi, Hiroko Makimoto

**Affiliations:** 1 Research Group for Safety and Health among Small and Medium-sized Enterprises, Japan Society for Occupational Health

**Keywords:** Japan, observational study, promotion of information, small and medium-sized enterprises, support organizations

The implementation of occupational health (OH) measures is insufficient among small and medium-sized enterprises (SMEs) in Japan^1)^. For small enterprises, the lack of a concrete image of OH measures has been identified as a major barrier to their implementation^2)^. The “Research Group for Safety and Health among Small and Medium-sized Enterprises, Japan Society for Occupational Health” published a collection of OH good practices, based on the results of an interview survey conducted among SMEs^3)^, on the website (the good practices [GP] website)^4)^ in 2022. However, SME employers may not be expected to access the GP website by themselves^5)^ for reasons such as being too busy and having limited interest in OH or health literacy. Thus, we planned to directly promote information on the GP website to SMEs through their support organizations (eg, chambers of commerce and industry, labor, and social security attorneys). This study aimed to investigate the effectiveness of this approach.

We recruited 65 support organizations and professionals of various types and sizes from SMEs located all over Japan using snowball sampling. Of these, 33 organizations participated in this project and were categorized into three types: i) OH service organizations and professionals (9 organizations and professionals), ii) economic organizations and professionals (4 organizations and professionals), and iii) other organizations and professionals (20 organizations and professionals). OH service organizations and professionals included occupational physicians, occupational health nurses, employee assistance program providers, and occupational health service organizations. Economic organizations and professionals included tax accountants, chambers of commerce and industry, and employer associations. Other organizations and professionals included labor and social security attorneys, insurance offices, publishing companies, administrative and academic organizations, and the healthcare industry. As this study did not use any personal information, the participating organizations and professionals were informed that the decision of participation was voluntary, and the interactions between the Research Group and organizations or people in the selected SMEs were within the common activities regarding the objective of the Group, our study was not regarded as the one subjected to the discussion in an ethical review board.

The intervention began in May 2023. We provided a message template for SMEs that encouraged recipients to visit the GP websites to support organizations and professionals. The full text of a message template is shown in eFigure 1. We requested that they send it to their associated SMEs on a separate day via email, websites, or as documents. We then confirmed the date of intervention for each organization and professional and plotted them according to the type of organization, shown as numbers 1 to 3 in Figure 1. Before (December 16, 2022, to May 15, 2023) and during the intervention period (May 16 to October 15, 2023), we counted the number of times the GP website was accessed.

**Fig. 1.**
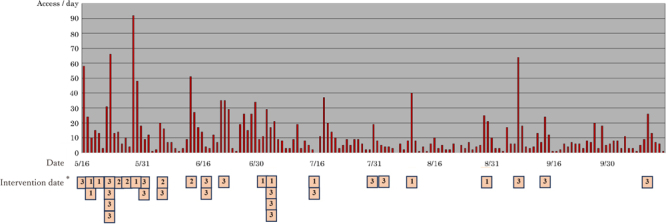
Number of accesses to the website in a day and date of sending messages to small and medium-sized enterprises from each organization and professional *Each number indicates the type of support organization and professional (below), and its coordinate position is the intervention date. 1) Occupational health service organizations and professionals 2) Economic organizations and professionals 3) Other organizations and professionals

Although the total number of times the GP website was accessed was only 234 in 5 months before baseline, it increased by approximately eight times (1,793 visits) in the 5 months after the intervention. Figure 1 shows the number of times the GP website was accessed per day and the date of sending messages to SMEs from each organization and professional. Immediately after the intervention by several organizations and professionals, the number of times the GP website was accessed increased to 50–100 per day.

This observational study conducted in Japan found that sending messages to SMEs through their support organizations and professionals drastically increased the number of times OH information was accessed. To the best of our knowledge, this is the first study to demonstrate the effectiveness of support organizations and professionals in promoting OH information for SMEs. The present findings show that several support organizations and professionals may have been influential in increasing the number of times OH information was accessed. Furthermore, not limited to OH service organizations and professionals, outreach to economic and other organizations and professionals seems to have great potential impact on expanding OH information to SMEs. For example, in Japan, labor and social security attorneys provide legal advisory services, including human resource management, to SMEs as legal consultants^6)^. Their total number is approximately 40,000, and they may have great potential for disseminating OH information to SMEs. A multichannel approach that includes various support organizations and professionals may be useful to promote information to SMEs^5)^. Thus, it is important to expand the networks.

This study has some limitations. First, we did not restrict access to the website, and the website may have been accessed by not only SMEs but also support organizations and professionals. Second, we recruited participants using snowball sampling, and this might have led to selection bias. Third, we did not know when an increase in access to the website occurred; that is, if interventions had been made at short intervals between support organizations and professionals, it may not have precisely shown the relationship between the intervention date and the number of times the website was accessed. Finally, we could not show the long-term behavior of accessing the website, mainly because of the relatively short intervention interval. In addition to short-term behavior, long-term behavior may be important for improving the promotion of OH information for SMEs. Caution should be exercised when interpreting the results. Further studies using questionnaire surveys must be conducted to determine the characteristics of those who access the GP site, assess how useful the GP site is, and identify what information they want.

## Supplementary Material

Supplementary eFigure 1
