# Supplementary material for: Promoting occupational health information in small and medium-sized enterprises in Japan
Source: Environ Occup Health Pract. 2025 Mar 18;7(1):2024-0014. doi: 10.1539/eohp.2024-0014 (PMC11960806; doi:10.1539/eohp.2024-0014)
Supplement: Supplementary file 1 — Supplementary eFigure 1 [file eohp-7-2024-0014-s001.pdf]

eFigure 1. A message template for SMEs*

We have posted a collection of good practices that are useful for SMEs

In 2021~2022, Research Group for Safety and Health among Small and Medium-sized Enterprises conducted interview surveys among SMEs about good practices (small ideas and initiatives) with the aim of clarifying hints and issues related to measures to promote the health and safety of employees. For helping SMEs, we summarized the good practices that had been carried out in the field and published them on the website (URL below).
https://www.oshsme.com/

Good practices are shown in the following four areas.
(1) COVID-19 countermeasures (see figure below)
(2) Measures against hazardous work
(3) Measures against lifestyle-related diseases
(4) Mental health measures

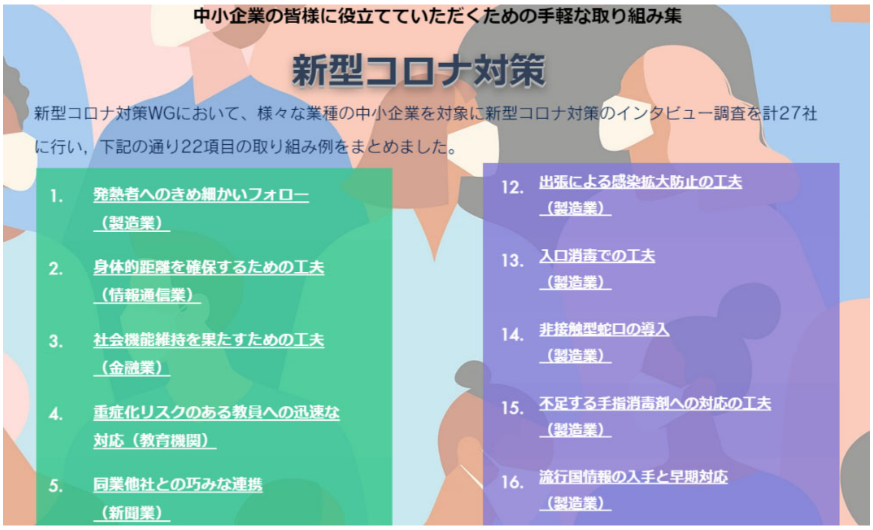

All viewing is free of charge. Please feel free to access it.

*SMEs: small and medium-sized enterprises
